# Supplementary material for: Capturing dynamic phage–pathogen coevolution by clinical surveillance
Source: Nature. 2026 Mar 11;653(8114):483–90. doi: 10.1038/s41586-026-10136-z (PMC12987554; doi:10.1038/s41586-026-10136-z)
Supplement: Supplementary file 1 — Supplementary Figs. 1–5. [file 41586_2026_10136_MOESM1_ESM.pdf]

nature portfolio

https://doi.org/10.1038/s41586-026-10136-z

Supplementary information

Capturing dynamic phage–pathogen coevolution by clinical surveillance

In the format provided by the authors and unedited

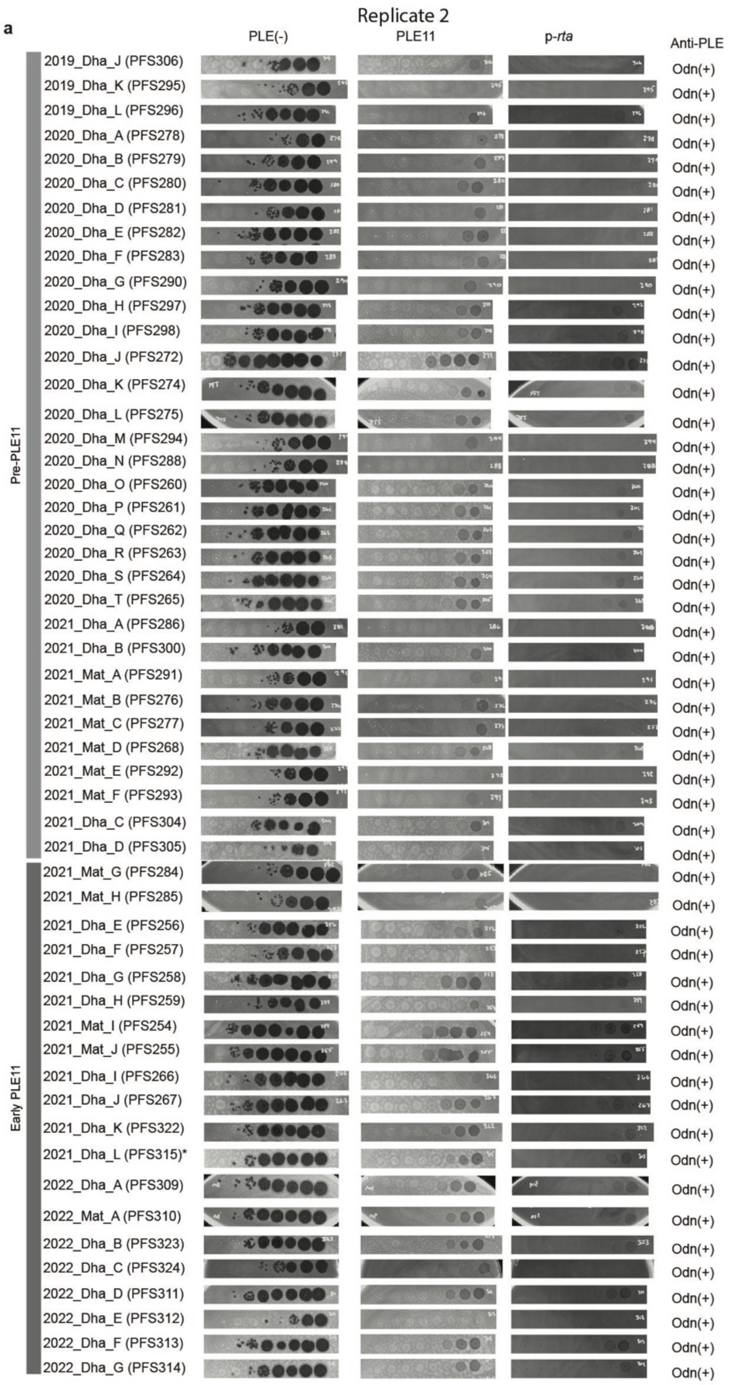

a
Replicate 2
PLE(-)
PLE11
p-rta
Anti-PLE
Pre-PLE11
Early PLE11
2019_Dha_J (PFS306) Odn(+)
2019_Dha_K (PFS295) Odn(+)
2019_Dha_L (PFS296) Odn(+)
2020_Dha_A (PFS278) Odn(+)
2020_Dha_B (PFS279) Odn(+)
2020_Dha_C (PFS280) Odn(+)
2020_Dha_D (PFS281) Odn(+)
2020_Dha_E (PFS282) Odn(+)
2020_Dha_F (PFS283) Odn(+)
2020_Dha_G (PFS290) Odn(+)
2020_Dha_H (PFS297) Odn(+)
2020_Dha_I (PFS298) Odn(+)
2020_Dha_J (PFS272) Odn(+)
2020_Dha_K (PFS274) Odn(+)
2020_Dha_L (PFS275) Odn(+)
2020_Dha_M (PFS294) Odn(+)
2020_Dha_N (PFS288) Odn(+)
2020_Dha_O (PFS260) Odn(+)
2020_Dha_P (PFS261) Odn(+)
2020_Dha_Q (PFS262) Odn(+)
2020_Dha_R (PFS263) Odn(+)
2020_Dha_S (PFS264) Odn(+)
2020_Dha_T (PFS265) Odn(+)
2021_Dha_A (PFS286) Odn(+)
2021_Dha_B (PFS300) Odn(+)
2021_Mat_A (PFS291) Odn(+)
2021_Mat_B (PFS276) Odn(+)
2021_Mat_C (PFS277) Odn(+)
2021_Mat_D (PFS268) Odn(+)
2021_Mat_E (PFS292) Odn(+)
2021_Mat_F (PFS293) Odn(+)
2021_Dha_C (PFS304) Odn(+)
2021_Dha_D (PFS305) Odn(+)
2021_Mat_G (PFS284) Odn(+)
2021_Mat_H (PFS285) Odn(+)
2021_Dha_E (PFS256) Odn(+)
2021_Dha_F (PFS257) Odn(+)
2021_Dha_G (PFS258) Odn(+)
2021_Dha_H (PFS259) Odn(+)
2021_Mat_I (PFS254) Odn(+)
2021_Mat_J (PFS255) Odn(+)
2021_Dha_I (PFS266) Odn(+)
2021_Dha_J (PFS267) Odn(+)
2021_Dha_K (PFS322) Odn(+)
2021_Dha_L (PFS315)* Odn(+)
2022_Dha_A (PFS309) Odn(+)
2022_Mat_A (PFS310) Odn(+)
2022_Dha_B (PFS323) Odn(+)
2022_Dha_C (PFS324) Odn(+)
2022_Dha_D (PFS311) Odn(+)
2022_Dha_E (PFS312) Odn(+)
2022_Dha_F (PFS313) Odn(+)
2022_Dha_G (PFS314) Odn(+)

a (continued)

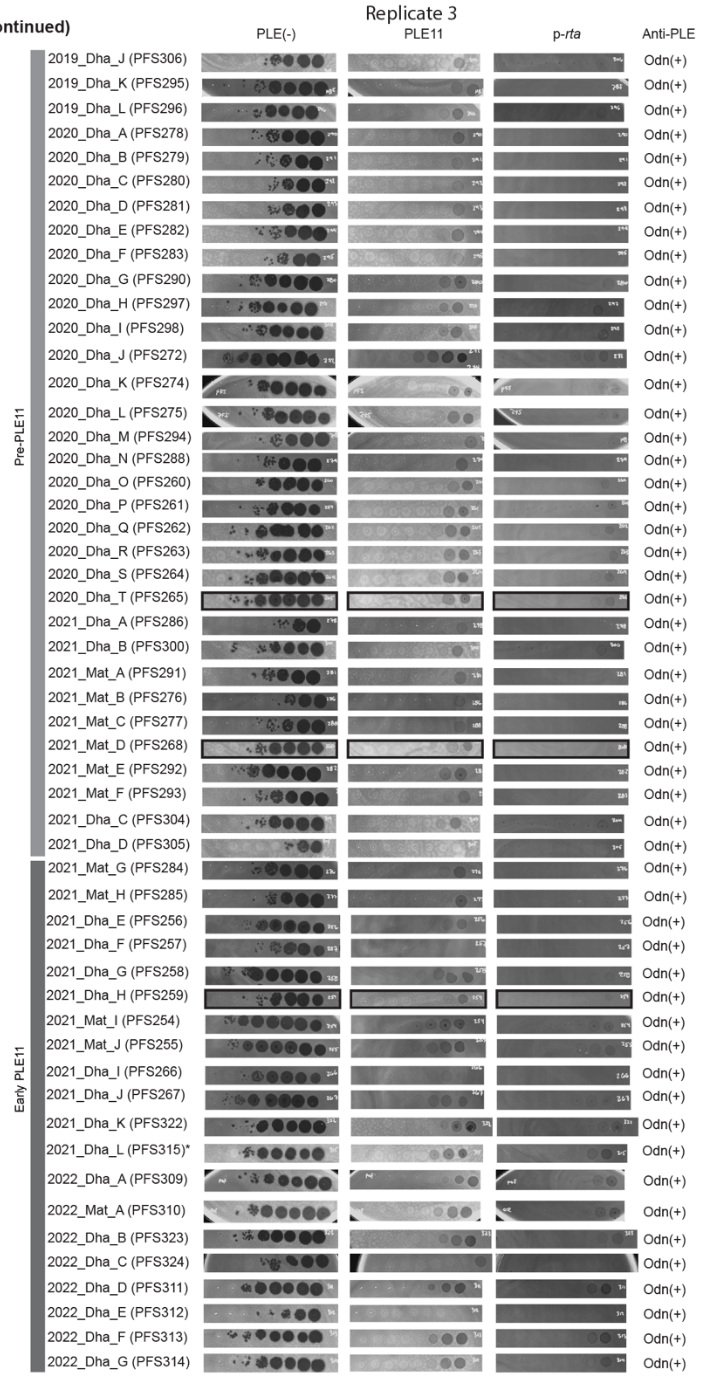

Replicate 3
PLE(-)
PLE11
p-rta
Anti-PLE
Pre-PLE11
2019_Dha_J (PFS306) Odn(+)
2019_Dha_K (PFS295) Odn(+)
2019_Dha_L (PFS296) Odn(+)
2020_Dha_A (PFS278) Odn(+)
2020_Dha_B (PFS279) Odn(+)
2020_Dha_C (PFS280) Odn(+)
2020_Dha_D (PFS281) Odn(+)
2020_Dha_E (PFS282) Odn(+)
2020_Dha_F (PFS283) Odn(+)
2020_Dha_G (PFS290) Odn(+)
2020_Dha_H (PFS297) Odn(+)
2020_Dha_I (PFS298) Odn(+)
2020_Dha_J (PFS272) Odn(+)
2020_Dha_K (PFS274) Odn(+)
2020_Dha_L (PFS275) Odn(+)
2020_Dha_M (PFS294) Odn(+)
2020_Dha_N (PFS288) Odn(+)
2020_Dha_O (PFS260) Odn(+)
2020_Dha_P (PFS261) Odn(+)
2020_Dha_Q (PFS262) Odn(+)
2020_Dha_R (PFS263) Odn(+)
2020_Dha_S (PFS264) Odn(+)
2020_Dha_T (PFS265) Odn(+)
2021_Dha_A (PFS286) Odn(+)
2021_Dha_B (PFS300) Odn(+)
2021_Mat_A (PFS291) Odn(+)
2021_Mat_B (PFS276) Odn(+)
2021_Mat_C (PFS277) Odn(+)
2021_Mat_D (PFS268) Odn(+)
2021_Mat_E (PFS292) Odn(+)
2021_Mat_F (PFS293) Odn(+)
2021_Dha_C (PFS304) Odn(+)
2021_Dha_D (PFS305) Odn(+)
Early PLE11
2021_Mat_G (PFS284) Odn(+)
2021_Mat_H (PFS285) Odn(+)
2021_Dha_E (PFS256) Odn(+)
2021_Dha_F (PFS257) Odn(+)
2021_Dha_G (PFS258) Odn(+)
2021_Dha_H (PFS259) Odn(+)
2021_Mat_I (PFS254) Odn(+)
2021_Mat_J (PFS255) Odn(+)
2021_Dha_I (PFS266) Odn(+)
2021_Dha_J (PFS267) Odn(+)
2021_Dha_K (PFS322) Odn(+)
2021_Dha_L (PFS315)* Odn(+)
2022_Dha_A (PFS309) Odn(+)
2022_Mat_A (PFS310) Odn(+)
2022_Dha_B (PFS323) Odn(+)
2022_Dha_C (PFS324) Odn(+)
2022_Dha_D (PFS311) Odn(+)
2022_Dha_E (PFS312) Odn(+)
2022_Dha_F (PFS313) Odn(+)
2022_Dha_G (PFS314) Odn(+)

Clinical PLE(-)

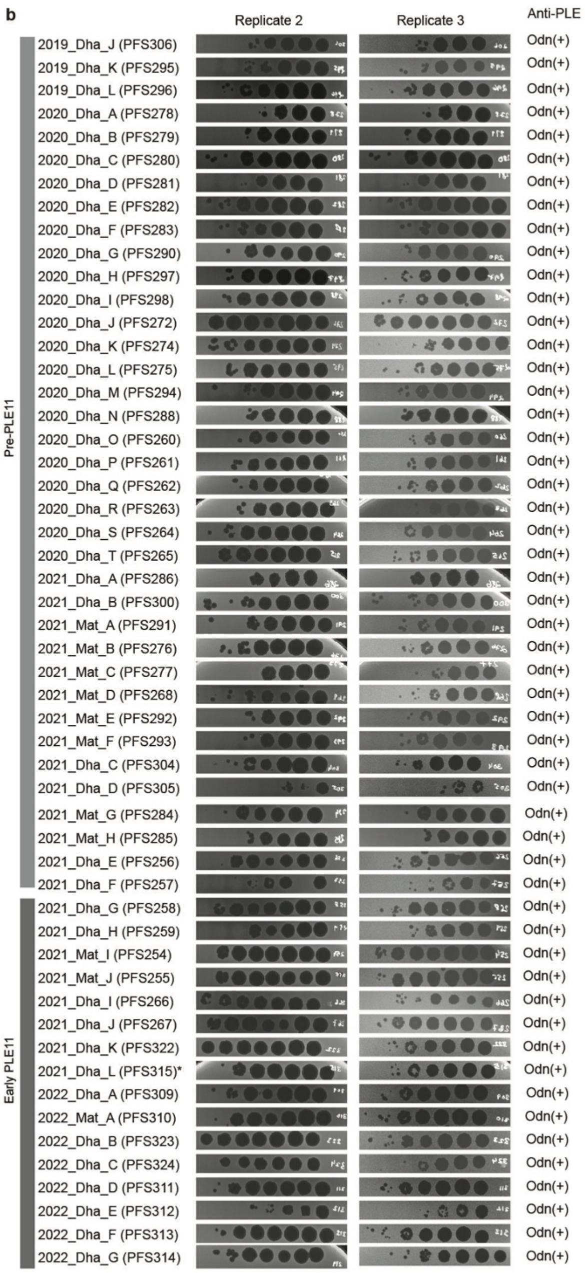

c

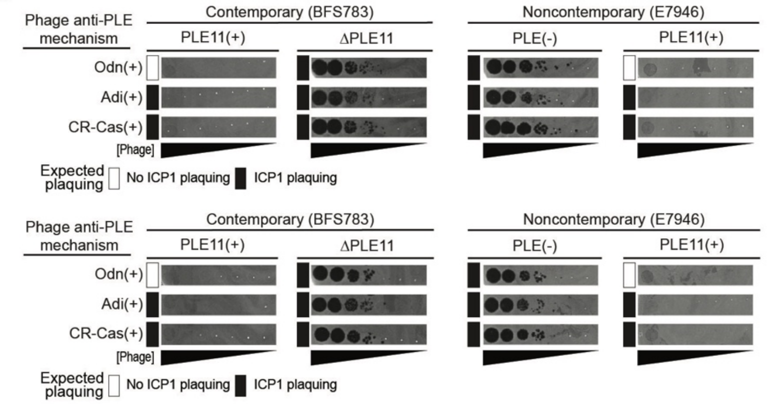

Supplementary Fig. 1. PLE11 protects *V. cholerae* from pre- and co-circulating Odn(+) phage isolates.
a) Biological replicates related to Extended Data Fig. 2. Plaquing of tenfold serially diluted ICP1 phage isolates from the pre-PLE11 and early-PLE11 periods (December 2019 to June 2022) on lawns of *V. cholerae* strain E7946, E7946 PLE11(+) and E7946 with a low-copy plasmid expressing PLE11 *rta* **(a)**, or on lawns of the clinical PLE(-) isolate BFS948 **(b)**. The gray background is the bacterial lawn, and the dark spots are zones of killing. Images that were used in the main text are indicated with black boxes. Standardized ICP1 names are shown (which include the year and location of isolation; Dha = Dhaka, Mat = Mathbaria), as well as lab designations (PFS#). The light gray shading indicates the period before PLE11 (pre-PLE11), the medium gray shading indicates the first year after the initial detection of PLE11 (early PLE11 period). All phage isolates from this period encode Odn as their sole anti-PLE mechanism; none of the whole genome sequenced isolates (n= 17) harbor substitutions in the TMP within the Rta-associated region (amino acid positions 314-387), but one isolate (marked by *) contains a TMP substitution (R132C) which does not provide resistance to PLE11 or Rta. Where isolates were genotyped by PCR for Odn vs CRISPR-Cas, the sequence of the *tmp* was not analyzed. **(c)** Replicates of spot plates for Fig. 1e. Plaquing of tenfold serially diluted ICP1 phage isolates with the anti-PLE mechanism indicated on lawns of *V. cholerae*. BFS783 refers to the first PLE11(+) clinical isolate identified in our surveillance (see arrow in Fig. 1c). E7946 PLE(-) and E7946 PLE11(+) are laboratory strains. The expected plaquing phenotype (based on validated infection outcomes from characterized PLEs) is indicated for each bacterial host-phage pair. The plaquing phenotype for Odn(+) phage on the noncontemporary (E7946) strain is representative of all ICP1 isolates recovered during the pre-PLE11 and early PLE11 periods (n = 53) (Extended Data Fig. 2a, Supplementary Fig. 1). CRISPR-Cas(+) and Adi(+) ICP1 isolates are historically collected isolates.

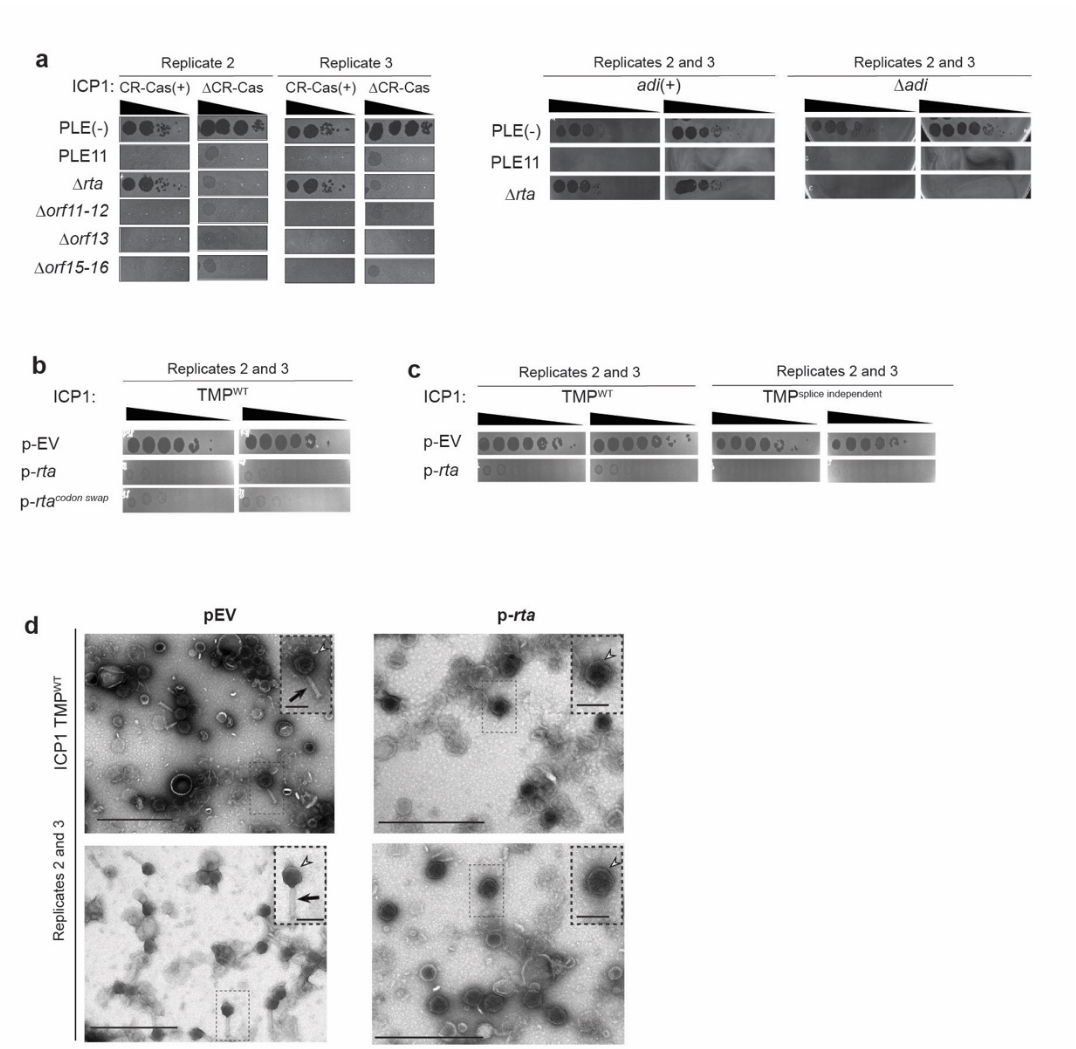

Supplementary Fig. 2 PLE11-encoded Rta restricts the assembly of tailed ICP1 virions

a) Plaquing of tenfold serially diluted ICP1 CR-Cas(+/-) (left, replicates of Fig. 2a) or ICP1 Adi(+/-) (right, replicates of Extended Data Fig. 3a) on lawns of *V. cholerae* strain E7946 and its PLE11(+) and PLE11 mutant derivatives. The gray background is the bacterial lawn, and the dark spots are zones of killing.

b) Replicates of Extended Data Fig. 3c showing plaquing of tenfold serially diluted ICP1 CR-Cas(-) on lawns of *V. cholerae* harboring an empty vector (pEV), a vector expressing Rta (p-*rta*), or a vector expressing codon-swapped *rta* (p-$rta^{codon\ swap}$).

c) Replicates of Extended Data Fig. 3d showing plaquing of tenfold serially diluted wild type (WT) ICP1 CR-Cas(-) wherein production of the tape measure protein (TMP) involves splicing (TMP^{WT}) or a mutant constructed to bypass splicing ($TMP^{splice\ independent}$) on lawns of *V. cholerae* harboring pEV or p-*rta.*

d) Replicates of Extended Data Fig. 3e Representative transmission electron micrographs (TEMs) of particles produced following ICP1 infection of PLE(-) *V. cholerae* with pEV or p-*rta* with inducer. The arrowheads indicate DNA-filled capsids, and the arrow indicates a tail. The scale bars are 500 nm and 100 nm for the zoomed-out and insets, respectively.

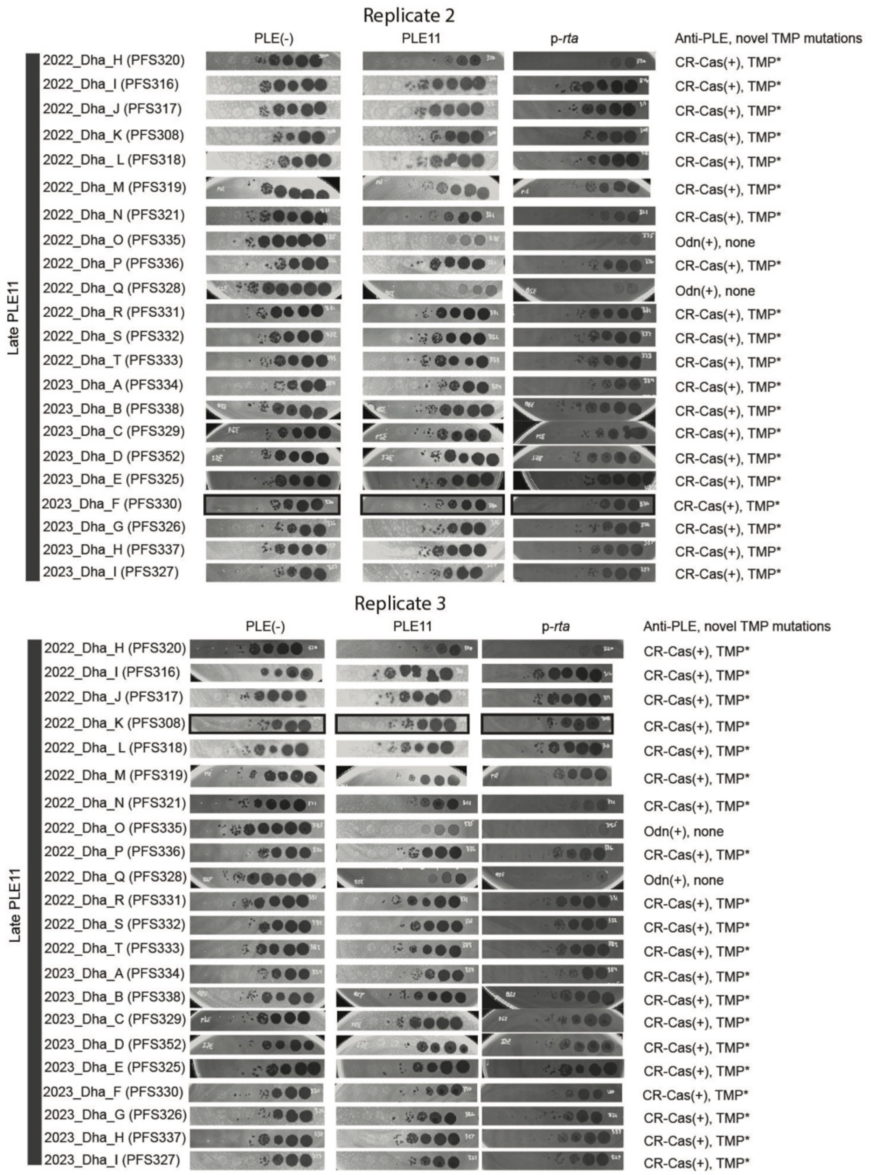

Supplementary Figure 3. ICP1 isolates from the late-PLE11 period have evolved to overcome PLE11-mediated defense and Rta

Biological replicates related to Extended Data Fig. 6. Plaquing of tenfold serially diluted ICP1 phage isolates from the late PLE11 period (July 2022 to December 2023) on lawns of *V. cholerae* strain E7946, E7946 PLE11(+) and E7946 with a low-copy plasmid expressing PLE11 *rta*. The gray background is the bacterial lawn, and the dark spots are zones of killing. Images that were used in the main text are indicated with black boxes. Standardized ICP1 names are shown (which include the year and location of isolation; Dha = Dhaka, Mat = Mathbaria), as well as lab designations (PFS#). The dark gray shading indicates the period after PLE11 (late-PLE11 period). Anti-PLE mechanisms and the presence of TMP mutations are indicated; TMP* refers to either substitution combination of L362P ± N355S, none indicates no substitutions.

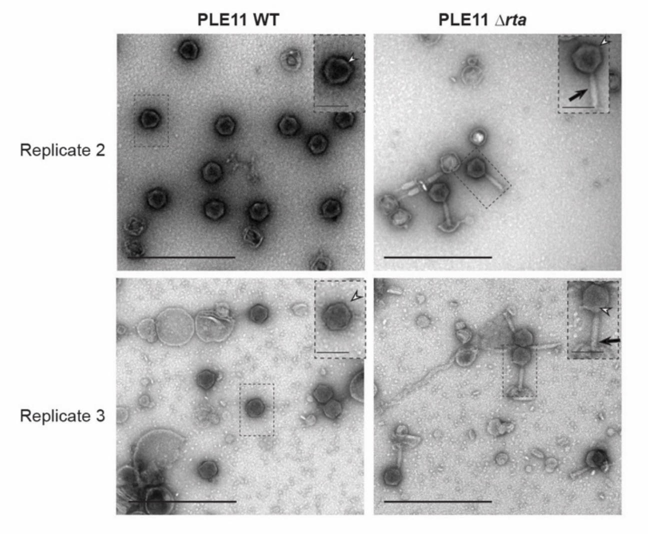

Supplementary Figure 4. Rta restricts tail assembly of CRISPR-Cas(+) ICP1
Replicates of Extended Data Fig. 9b showing representative transmission electron micrographs (TEMs) of particles produced following CRISPR-Cas(+) ICP1 infection of PLE11 WT or Δ*rta V. cholerae.* The arrowheads indicate DNA-filled capsids, and the arrow indicates a tail. The scale bars are 500 nm and 100 nm for the zoomed-out and insets, respectively.

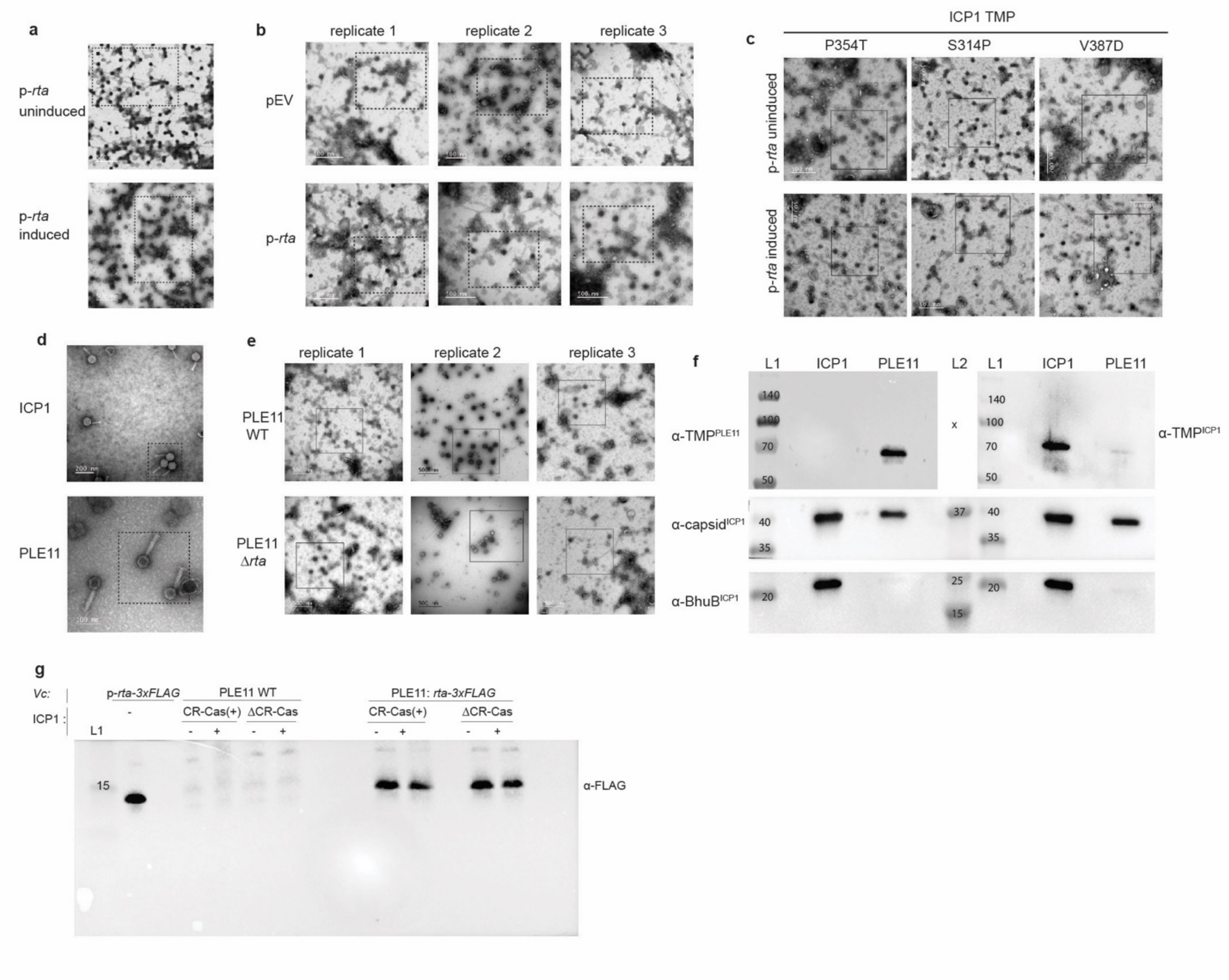

Supplementary Figure 5. Uncropped transmission electron micrographs and western blots
a-e) Uncropped transmission electron micrographs (TEMs) with dashed boxes indicating the regions selected for presentation in the main text in Fig. 2d **(a)**, Extended data Fig. 3e & Supplementary Fig. 2d **(b)**, Extended data 3f **(c)**, Fig. 4a **(d)**, and Extended Data Fig. 9b & Supplementary Fig. 4 **(e)**. Scale bars recorded at the original acquisition are shown. **f)** Uncropped western blots used for Fig. 4d. L1 and L2 indicate ladder 1 and ladder 2, respectively, and the size in kDa is noted on the blots. Antibodies used are indicated. ICP1 and PLE indicate samples of purified virions analyzed in this experiment. **g)** Uncropped western blot used to generate Extended data Fig. 9a. The genotypes of *V. cholerae* and ICP1 strains used in the experiment are indicated. The + and – signs indicate sample collection done prior to the addition of phage and 16 minutes post-infection, respectively. L1 indicates the ladder, and the size in kDa is noted.
