# Supplementary material for: Capturing dynamic phage–pathogen coevolution by clinical surveillance
Source: Nature. 2026 Mar 11;653(8114):483–90. doi: 10.1038/s41586-026-10136-z (PMC12987554; doi:10.1038/s41586-026-10136-z)
Supplement: Supplementary file 2 — Reporting Summary [file 41586_2026_10136_MOESM2_ESM.pdf]

nature portfolio

Corresponding author(s): Kimberley Seed

Last updated by author(s): Jan 8, 2026

Reporting Summary

Nature Portfolio wishes to improve the reproducibility of the work that we publish. This form provides structure for consistency and transparency in reporting. For further information on Nature Portfolio policies, see our Editorial Policies and the Editorial Policy Checklist.

Statistics

For all statistical analyses, confirm that the following items are present in the figure legend, table legend, main text, or Methods section.

n/a	Confirmed	
☐	☒	The exact sample size (*n*) for each experimental group/condition, given as a discrete number and unit of measurement
☐	☒	A statement on whether measurements were taken from distinct samples or whether the same sample was measured repeatedly
☐	☒	The statistical test(s) used AND whether they are one- or two-sided<br>*Only common tests should be described solely by name; describe more complex techniques in the Methods section.*
☒	☐	A description of all covariates tested
☒	☐	A description of any assumptions or corrections, such as tests of normality and adjustment for multiple comparisons
☐	☒	A full description of the statistical parameters including central tendency (e.g. means) or other basic estimates (e.g. regression coefficient) AND variation (e.g. standard deviation) or associated estimates of uncertainty (e.g. confidence intervals)
☐	☒	For null hypothesis testing, the test statistic (e.g. *F*, *t*, *r*) with confidence intervals, effect sizes, degrees of freedom and *P* value noted<br>*Give P values as exact values whenever suitable.*
☒	☐	For Bayesian analysis, information on the choice of priors and Markov chain Monte Carlo settings
☒	☐	For hierarchical and complex designs, identification of the appropriate level for tests and full reporting of outcomes
☒	☐	Estimates of effect sizes (e.g. Cohen's *d*, Pearson's *r*), indicating how they were calculated

Our web collection on statistics for biologists contains articles on many of the points above.

Software and code

Policy information about availability of computer code

Data collection	no software was used for data collection
Data analysis	Sequencing data was analyzed with fastp v0.23.2, Spades v3.15.4, snippy v4.6.0, IQ-TREE v2.2.0, ModelFinder, ViPTree version 4.0, tBLASTx version 2.17.0, VIRIDICweb, NCBI BLAST, and HHPred. Structural predictions were made using ColabFOLD on COSMIC2, and structural visualizations were done using UCSF ChimeraX. Genome visualizations were generated using Clinkerv0.0.28 and R 4.4., using the gggenes package.

For manuscripts utilizing custom algorithms or software that are central to the research but not yet described in published literature, software must be made available to editors and reviewers. We strongly encourage code deposition in a community repository (e.g. GitHub). See the Nature Portfolio guidelines for submitting code & software for further information.

Data

Policy information about availability of data

All manuscripts must include a data availability statement. This statement should provide the following information, where applicable:

- Accession codes, unique identifiers, or web links for publicly available datasets
- A description of any restrictions on data availability
- For clinical datasets or third party data, please ensure that the statement adheres to our policy

Sequence data for V. cholerae and ICP1 isolates from clinical samples and for ICP1 escape phages have been deposited in the NCBI Sequence Reads Archive (SRA)

under BioProject PRJNA1195958. The PLE11 sequence has been deposited to GenBank (accession PQ783903). The mass spectrometry proteomics data have been deposited to the ProteomeXchange Consortium via the PRIDE partner repository with the dataset identifier PXD058665 under the DOI 10.6019/PXD058665. Publicly available sequence data used in this study with metadata and accession numbers are provided in Supplementary Table 9. Publicly available datasets were used in structural comparisons (PDB:51R0, PDB:2OB9). All other data supporting the findings of this study are available in the paper and Supplementary Information. For gel and TEM image source data, see Supplementary Fig. 5. Source data are provided with this paper.

Research involving human participants, their data, or biological material

Policy information about studies with human participants or human data. See also policy information about sex, gender (identity/presentation), and sexual orientation and race, ethnicity and racism.

Reporting on sex and gender	this section is not relevant as the study did not focus on human cholera patients but on the bacterial and phage isolates from their stool; no human material was collected or studied
Reporting on race, ethnicity, or other socially relevant groupings	this section is not relevant as the study did not focus on human cholera patients but on the bacterial and phage isolates from their stool; no human material was collected or studied
Population characteristics	this section is not relevant as the study did not focus on human cholera patients but on the bacterial and phage isolates from their stool; no human material was collected or studied
Recruitment	Stool samples were collected from suspected cholera patients at the icddr,b Dhaka Hospital and the Government Health Complex in Mathbaria, Pirojpur, under protocol number PR-16083 approved by the icddr,b Ethical Review Committee, with written consent obtained from participants or their guardians.
Ethics oversight	Dr. Alam's protocol number PR-16083 is approved by the icddr,b Ethical Review Committee (ERC); Dr. Seed is not required to have an IRB as all samples have been de-identified

Note that full information on the approval of the study protocol must also be provided in the manuscript.

Field-specific reporting

Please select the one below that is the best fit for your research. If you are not sure, read the appropriate sections before making your selection.

[x] Life sciences [ ] Behavioural & social sciences [ ] Ecological, evolutionary & environmental sciences

For a reference copy of the document with all sections, see nature.com/documents/nr-reporting-summary-flat.pdf

Life sciences study design

All studies must disclose on these points even when the disclosure is negative.

Sample size	Sample size was not calculated for comparative genomic studies because no standard or statistical approach for choosing the appropriate number of strains has been established. For all quantitative experiments and phenotypic observations using phage spot plates and electron microscopy, we chose to replicate experiments in biological triplicate as is routine to indicate reproducibility and allow for statistical analysis. Sample sizes for particle counting using EM images were determined by the density of particles on the grids generated from three biological replicates of particle purifications. TEMs of ICP1 with TMP substitutions were done one time each on three independent substitution mutants. The proteomics analyses of purified ICP1 and PLE11 particles were conducted once due to the high time and monetary costs, but results were corroborated through independent means (i.e. alterations to the structural composition of PLE particles obtained by an independent preparation of particles as assessed by western blot in Fig. 4d). Western blotting of Rta-3X FLAG was done one time which incorporated two biological replicates of the uninfected sample which was the key finding regarding Rta's expression prior to phage infection.
Data exclusions	No data were excluded from the analysis.
Replication	All reported findings were successfully reproduced through independent biological replicates or corroborating analyses as described above.
Randomization	No experimental groups or control groups were subjectively chosen therefore no randomization is required.
Blinding	Blinding is not relevant to the experimental design as all data were obtained objectively.

Reporting for specific materials, systems and methods

We require information from authors about some types of materials, experimental systems and methods used in many studies. Here, indicate whether each material, system or method listed is relevant to your study. If you are not sure if a list item applies to your research, read the appropriate section before selecting a response.

Materials & experimental systems

n/a	Involved in the study
☐	☒ Antibodies
☒	☐ Eukaryotic cell lines
☒	☐ Palaeontology and archaeology
☒	☐ Animals and other organisms
☒	☐ Clinical data
☒	☐ Dual use research of concern
☒	☐ Plants

Methods

n/a	Involved in the study
☒	☐ ChIP-seq
☒	☐ Flow cytometry
☒	☐ MRI-based neuroimaging

Antibodies

Antibodies used

Custom antibodies generated in rabbits by GenScript were used for anti-ICP1 baseplate hub (BhuB) (PolyExpress Premium Cat. no. SC1676), anti-ICP1 tape measure protein (TMP) (PolyExpress Premium Cat. no. SC1676), anti-PLE11 tape measure protein (TMP) (PolyExpress Premium Cat. no. SC1676) and anti-ICP1 capsid (PolyExpress Gold cat. no. SC1649). Anti-FLAG antibody was purchased through GenScript (cat. No. A00170-40).

Validation

Custom Antibodies were validated by the manufacturer using ELISA and western blot analyses of purified antigen.
Anti-FLAG: validated by "Western blot analysis of DYKDDDDK tagged fusion proteins expressed in E. coli cell lysate" Additionally, we included positive and negative controls in our western blotting for validation of the detection of FLAG tagged RTA (in ED Figure 9, positive control was plasmid expressing rta 3xFLAG and untagged PLE11 strain was used as negative control).

Plants

Seed stocks

n/a

Novel plant genotypes

n/a

Authentication

n/a
